# Nomogram for Predicting Persistent Organ Failure With Acute Pancreatitis in Pregnancy

**DOI:** 10.3389/fendo.2022.863037

**Published:** 2022-04-14

**Authors:** Chengcheng Sheng, Zongxu Xu, Jun Wang

**Affiliations:** Department of Obstetrics and Gynecology, Shengjing Hospital of China Medical University, Shenyang City, China

**Keywords:** gestation, acute pancreatitis (AP), hypertriglyceridemia (HTG), persistent organ failure, nomogram model

## Abstract

**Background:**

Acute pancreatitis in pregnancy (APIP) with persistent organ failure (POF) poses a high risk of death for mother and fetus. This study sought to create a nomogram model for early prediction of POF with APIP patients.

**Methods:**

We conducted a cross-sectional study on APIP patients with organ failure (OF) between January 2012 and March 2021. 131 patients were collected. Their clinical courses and pregnancy outcomes were obtained. Risk factors for POF were identified by univariate and multivariate logistic regression analysis. Prediction models with POF were built and nomogram was plotted. The performance of the nomogram was evaluated by using a bootstrapped-concordance index and calibration plots.

**Results:**

Hypertriglyceridemia was the most common etiology in this group of APIP patients, which accounted for 50% of transient organ failure (TOF) and 72.3% of POF. All in-hospital maternal death was in the POF group (*P*<0.05), which also had a significantly higher perinatal mortality rate than the TOF group (*P*<0.05). Univariate and multivariate logistic regression analysis determined that lactate dehydrogenase, triglycerides, serum creatinine, and procalcitonin were independent risk factors for predicting POF in APIP. A nomogram for POF was created by using the four indicators. The area under the curve was 0.875 (95%CI: 0.80–0.95). The nomogram had a bootstrapped-concordance index of 0.85 and was well-calibrated.

**Conclusions:**

Hypertriglyceridemia was the leading cause of organ failure-related APIP. Lactate dehydrogenase, triglycerides, serum creatinine, and procalcitonin were the independent risk factors of POF in APIP. Our nomogram model showed an effective prediction of POF with the four indicators in APIP patients.

## Introduction

Acute pancreatitis (AP) is one of the common diseases of gastroenterology and the incidence of AP is about 13-45/100,000 in the general population (1, 2). Severe acute pancreatitis (SAP) accounts for 5-20% of the entire AP and is an acute abdomen with a hazardous condition, many complications, and a high mortality rate (3). The reported incidence of AP among pregnant women is 1/1000–1/12000 (1, 4), which is higher than that in the general population. Due to the unique physiological state of pregnancy, abdominal pain and other clinical manifestations of acute pancreatitis in pregnancy (APIP) patients are often easily masked, leading to misdiagnosis. APIP usually develops rapidly. Once treatment is delayed, it will progress to SAP, which may lead to poor prognosis and even endanger maternal and fetal life (5).

SAP is defined by the presence of persistent organ failure [POF, organ failure (OF)≧48 h]. SAP in pregnancy (SAPIP) is hazardous to the health of pregnant women, and the maternal mortality rate can be as high as about 20-40% (1, 6). The fetus of SAPIP is even prone to intrauterine distress and death (7–9). Previous studies showed that the prognosis of mothers and fetus were improved in patients with transient organ failure (TOF, OF less than 48h) (3). Early recognition of patients at risk of persistent organ failure is critical to initiate high-dependency or intensive care treatment, and reduce morbidity and mortality. Many studies have been carried out on the early prediction of POF (7, 10–12), while few reports focused on the prediction of POF in APIP patients.

In this study, we collected data from APIP patients with OF treated in the Shengjing Hospital of China Medical University in the past 9 years and analyzed the clinical features, clinical course, and maternal and perinatal outcomes. The aim was to identify clinical indicators that can effectively predict POF upon patient admission. Results from this study could help identify patients with higher risks of early POF and support early implementation of close monitoring and active treatment for these patients.

## Methods

### Patients Selection

**Figure 1:**
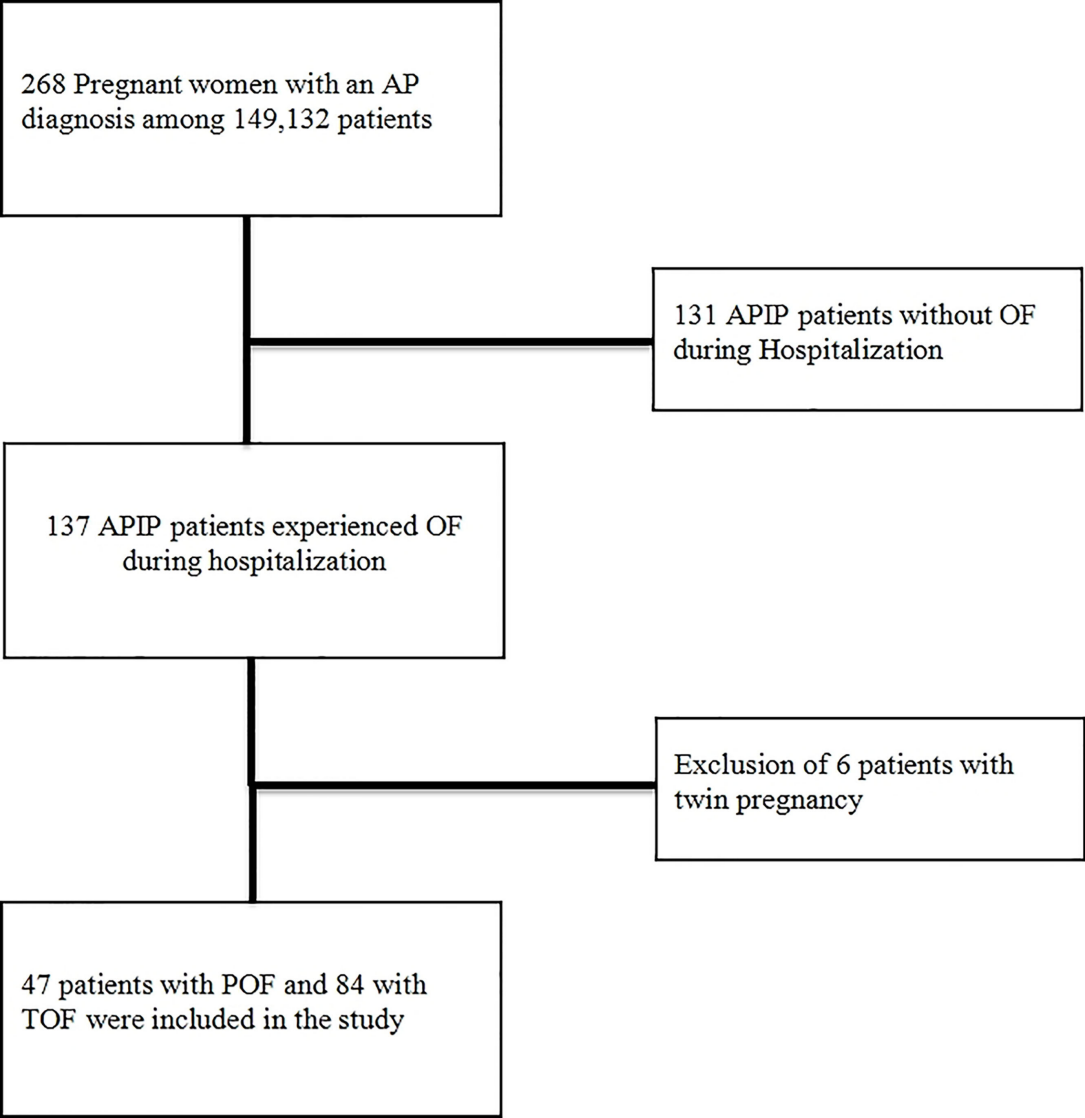
The selection process for patients in a flow chart. AP, acute pancreatitis; APIP, Acute pancreatitis in pregnancy; OF, organ failure; POF, persistent organ failure; TOF, transient organ failure.

As shown in a flow chart (**Figure 1**), among 149,132 pregnant women hospitalized from January 1, 2012 to March 31, 2021 in a tertiary hospital of the Northeast China, the Shengjing Hospital of China Medical University, 268 APIP patients were automatically screened through the electronic medical record system. After reviewing the electronic medical records, 131 APIP patients without OF and 6 twin APIP patients with OF were excluded, and 131 singleton APIPs with OF were included in the present study.

### Data Collection

Clinical data were collected from the electronic medical database of the Shengjing Hospital of China Medical University, which included demographic characteristics, relevant medical history, pregnancy history, comorbidities, imaging results, and laboratory examination findings. Neonatal survival outcomes were followed up to 7 days after birth and maternal survival outcomes were followed up until discharge.

### Relevant Definitions

The APIP diagnosis was based on revised 2012 revision of the Atlanta criteria, patients meeting ≥2 out of the following criteria were diagnosed with APIP: ① abdominal pain characteristic of AP; ② serum amylase or lipase greater than 3 times the upper limit of normal; ③ findings on cross-sectional abdominal imaging consistent with acute pancreatitis (3).

The severity classification of APIP was graded referring to 2012 revision of the Atlanta criteria. Mild acute pancreatitis (MAP) referred to pancreatitis without organ dysfunction or generalized complications; AP patients were diagnosed as moderately severe acute pancreatitis (MSAP) if they had at least one of the following features: TOF (OF< 48 h), deterioration of former comorbidities disease or local complications including acute peri-pancreatic fluid collections, pseudocysts, acute necrotic collections, and walled-off necrosis; patients were diagnosed as SAP if they experienced POF (OF≧48 h) (3).

OF was defined based on the modified Marshall scoring: circulatory failure, systolic blood pressure <90 mmHg with no fluid response; respiratory failure, PaO2/FiO2 ≤300; renal failure, blood serum creatinine (SCr) ≥170 μmol/L (13).

Biliary pancreatitis: alanine aminotransferase (ALT) >150 U/L within 48h of admission and biliary lesions on abdominal ultrasonography or magnetic resonance cholangiopancreatography (12).

Hypertriglyceridemic pancreatitis (HTGP): serum triglycerides (TG) ≥11.3 mmol/L or between 5.65 mmol/L-11.3 mmol/L with lipid turbidity after excluding biliary, alcohol, or medication factors (14).

Idiopathic pancreatitis: was defined as a form of disease with a similar presentation to SAPIP based on a radiological diagnosis after excluding biliary, alcohol, hypertriglyceridemia (HTG), medication, trauma, autoimmune, and surgical factors (15).

Diabetic ketoacidosis (DKA) was confirmed if all the following criteria were met: (1) blood glucose >13.9 mmol/L; (2) arterial blood pH <7.3, serum HCO3 ≤18 mmol/L; (3) Urine or serum ketone positive (16).

### Ethics and Funding

This study obtained institutional ethical approval from the ethics committee of the Shengjing Hospital of China Medical University (Approval No 2019PS683K) and complied with the Declaration of Helsinki. This study was supported by the Natural Science Foundation of the Liaoning Province (20180530076). The supporters of this work had no involvement in the study design; collection, analysis, and interpretation of the data; writing of the report; or decision to submit the article for publication.

### Statistics

Statistical analyses were performed using SPSS 26.0 software (IBM Corp., Armonk, NY) and R statistical software (R Foundation for Statistical Computing, Lanzhou, China) (17). The sample size was > 100. The Kolmogorov-Smirnov test for normality was used to estimate data distribution. Normally distributed data are expressed as mean ± standard deviation $\left(\text{i}.\text{e}.,\bar{X}\pm \text{SD}\right)$ and one-way analysis of variance was used for between-group comparisons. Non-normally distributed data are expressed as median (interquartile range) and non-parametric tests were used for between-group comparisons. Chi-squared tests were utilized to analyze count data. P<0.05 indicated statistical significance.

Univariate logistic regression analysis was conducted to identify risk factors for POF. Risk factors with P value < 0.05 in univariate analysis were included in a multivariate analysis. Multivariate logistic regression analysis was performed to identify independent risk factors, and a stepwise method was used to identify the useful combination of factors that could predict POF. A nomogram for POF was created based on the multivariate logistic regression model. The performance of the nomogram was evaluated using a concordance index (C-index) and calibration plots with bootstrap samples. A C-index is a numerical measure of discriminative ability and calibration curves are graphic evaluations of predictive ability that compare observed probabilities with nomogram-predicted probabilities. The nomogram was constructed using the “rms” package (18).

## Results

### General Feature of APIP Patients With Organ Failure

In this group of APIP with OF patients, their age range was 19–45 years. The disease onset range was 11–40 gestational weeks, with 2 patients in the first trimester (before 14 weeks), 20 in the second trimester (14-27+6 weeks), and 109 in the third trimester (after 28 weeks). The etiology of APIP patients with OF was HTG in 76 patients (58.0%), biliary in 20 (15.3%), and idiopathic in 35 (26.7%).

96 patients (72.3%) presented with upper abdominal pain and 51 (38.9%) were presented with nausea and vomiting. 18 patients (13.7%) exhibited overeating before disease onset. 39 patients (29.8%) sought medical help within 12 h of symptom onset, while 58 (44.2%) sought medical help within 12–24 h. The mean length of hospitalization was 19.45 ± 14.53 days (2–117 days) and 124 patients (94.5%) received intensive care.

### General Condition Between TOF and POF Groups

All the enrolled patients were categorized into TOF and POF groups and the general condition was compared between the two groups. The average age of the POF group was older than that of the TOF group (P=0.015). There were significant differences in the distribution of trimesters of pregnancy between POF and TOF groups (P=0.040). The etiological composition of the two groups was significantly different (P= 0.045). The ratio of biliary in TOF group was higher than that of the POF group (17.9% VS 10.6%), while the POF group has more HTGP than the TOF group (72.3% VS 50%).

There were no significant differences in body mass index (BMI), gravidity, parity, initial symptoms, and history of diabetes and hypertension between the two groups of patients (**Table 1**).

**Table 1:** Characteristics of APIP patients between POF and TOF groups.

	TOF (n=84) (n=81)	POF (n=47) (n=23)	*P* value
Age, years	28.9 ± 4.9	31.3 ± 5.7	0.015^#^
BMI, kg/m^2^	27.1 ± 3.5	27.4 ± 3.7	0.680
*Gravidity	2 (1-3)	2 (1-3)	0.325
*Parity	0 (0-1)	0 (0-1)	0.800
Trimester of pregnancy, n (%)			0.040^#^
First (before 14 weeks)	1 (1.2)	1 (2.1)	
Second (14-27^+6^ weeks)	8 (9.5)	12 (25.5)	
Third (after 28 weeks)	75 (89.3)	34 (72.3)	
Etiology, n (%)			0.045^#^
Biliary (n=59)	15 (17.9)	5 (10.6)	
HTG	42 (50.0)	34 (72.3)	
Idiopathic	27 (32.1)	8 (17.0)	
Initial symptoms, n(%)			
Upper abdominal pain	61 (72.6)	35 (74.5)	0.819
Vomiting	34 (40.5)	17 (36.2)	0.628
Diabetes, n (%)			0.208
Diabetes in pregnancy	25 (29.8)	19 (40.4)	
Gestational diabetes	1 (1.2)	2 (4.3)	
Hypertensive disorders, n (%)			0.215
Gestational hypertension	4 (4.8)	6 (12.8)	
Preeclampsia	3 (3.6)	6 (12.8)	
Eclampsia	3 (3.6)	0 (0)	

“*”indicates that data were not normally distributed and are expressed as median (interquartile range), M(Q). Non-parametric tests were used for between-group comparisons; the normally distributed data are expressed as mean (standard deviation), $\bar{X}\pm \text{SD}.$

One-way analysis of variance was used for between-group comparisons. Chi-squared tests were utilized to analyze count data.

“#”indicates the difference was statistically significant.

APIP, acute pancreatitis in pregnancy; BMI, body mass index; HTG, hypertriglyceridemia; POF, persistent organ failure; TOF, transient organ failure.

### Clinical Course of APIP Patient With TOF and POF

All patients developed OF within 1 week of onset. Respiratory failure is the commonest OF. There was no significant difference in the incidence of renal failure between the two groups. The incidence of respiratory failure and circulatory failure was significantly higher in the POF group than that in the TOF group (P=0.006 and P=0.001, respectively). Higher total hospital costs, longer hospital and intensive care unit (ICU) stay were noted among the patients in the POF group compared to the patients in the TOP group (P<0.05).

Detailed analyses revealed that the incidences of hyperglycemia (P=0.013), hypocalcemia (P=0.002), and DKA (P=0.001) were significantly higher in the POF group. Although the incidence of pleural (P=0.003) and peritoneal effusions (P=0.012) were also significantly higher in the POF group, the incidence of pelvic effusion was not (**Table 2**).

**Table 2:** Comparison of the clinical course between POF and TOF groups.

	TOF (n=84) (n=59)	POF (n=47) (n=23)	*P* value
*Length of hospital stay, days	14 (9-19)	21 (16-34)	0.001^#^
*Length of ICU stay, days	3 (2-5)	7 (4-12)	<0.05^#^
*Total costs, ¥	64496(44532-846211)	112942(74235-222541)	<0.05^#^
Interval of onset to OF	4 (3-6)	4 (3-7)	0.213
OF, n (%)			
Circulatory failure	22 (26.2)	26 (55.3)	0.001^#^
Respiratory failure	46 (54.8)	37 (78.7)	0.006^#^
Renal failure	26 (31.0)	17 (36.2)	0.542
complications			
SIRS, n (%)	45 (53.6)	34 (72.3)	0.036^#^
DKA, n (%)	9 (10.7)	19 (40.4)	0.001^#^
Pleural effusion, n (%)	36 (42.9)	33 (70.2)	0.003^#^
Peritoneal effusion, n (%)	38 (45.2)	32 (68.1)	0.012^#^
Pelvic effusion, n (%)	22 (26.2)	19 (40.4)	0.092
Fatty liver disease, n (%)	20 (28.2)	8 (19.5)	0.308
Hyperglycemia, n (%)	46 (54.8)	36 (76.6)	0.013^#^
Hypocalcemia, n (%)	22 (26.2)	25 (53.2)	0.002^#^
Hypokalemia, n (%)	19 (22.6)	11 (23.4)	0.918
Hyponatremia, n (%)	56 (66.7)	32 (68.1)	0.868
Localized complications, n (%)			0.058
Acute peri-pancreatic fluid collectionfluid collection	28 (33.3)	17 (36.2)	
Pseudocyst	16 (19.0)	4 (8.5)	
Walled-off necrosis	3 (3.6)	8 (17.0)	
Acute necrotic collections	2 (2.4)	3 (6.4)	
Mode of delivery, n (%)			0.415
Vaginal delivery	10 (11.9)	8 (17.0)	
Cesarean birth	74 (88.1)	39 (83.0)	
Pregnancy outcome, n (%)			0.015^#^
Abortion	11 (13.1)	12 (25.5)	
Premature birth	57 (67.9)	32 (68.1)	
Term birth	16 (19.0)	3 (6.4)	
Maternal deaths, n (%)	0 (0)	7 (14.9)	<0.05^#^
Perinatal loss, n (%)	19 (22.6)	29 (61.7)	<0.05^#^

“*”indicates that data were not normally distributed and are expressed as median (interquartile range), M(Q). Chi-squared tests were utilized to analyze count data.

“#”indicates the difference was statistically significant.

[Hyperglycemia was defined as fasting blood glucose ≥7.8 mmol/L; hypocalcemia was defined as serum Ca2+ <7.5 mg/dL (1.88 mmol/L); hypokalemia was defined as serum K+ <3.5 mmol/L; hyponatremia was defined as serum Na+ <135 mmol/L].

APIP, acute pancreatitis in pregnancy; DKA, diabetic ketoacidosis; HTG, hypertriglyceridemia; ICU, intensive care unit; OF, organ failure; POF, persistent organ failure; SIRS, systemic inflammatory response syndrome; TOF, transient organ failure.

### Maternal and Pregnancy Outcomes

All 131 patients delivered during hospitalization. Gestation was terminated by different approaches, mainly based on fetal and maternal conditions, with cesarean section being the main mode of delivery in the two groups (**Table 2**).

7 maternal deaths were observed in the present study, they were all with HTG as etiology and from the POF group (14.9%, P<0.05, **Table 2**). 3 died from multiple organ dysfunctions (at gestational week 34, 23, and 34, respectively), 2 from cardiac sudden death (at gestational week 32 and 33), 1 from hypoxic encephalopathy (at gestational week 25) and 1 from hemorrhagic shock (at gestational week 30). Seven maternal deaths were all happened during the hospitalization.

The perinatal loss rate was 61.7% in the POF group which was significantly higher than that in the TOF group (22.6%, P<0.05, **Table 2**).

### Perinatal Outcomes

83 (63.3%) neonates were born alive in the present study. None of the fetuses of those mothers with disease onset in the first and second trimesters survived. **Table 3** showed the neonatal characteristics. The gestational age was significantly younger in the POF group than that in the TOF group. There were no significant differences between the POF and TOF groups in the remaining neonatal characteristics.

**Table 3:** Comparison of living neonates between POF and TOF groups.

	TOF (n=63) (n=59)	POF (n=20) (n=23)	*P* value
*Gestational age, weeks	34 (32-36)	33 (30-35)5)	0.006^#^
Weight, g	2486 ± 785	2301 ± 797.67	0.330
*Length, cm	47 (43-50)	47 (44-50)	0.664
*Head circumference, cm	33 (31-35)	33 (28-34)	0.658
*Chest circumference, cm	31(29-34)	30 (26-32)	0.293
1 min Apgar score, n (%)			0.655
0-3	3 (4.7)	1(5.6)	
4-7	18 (28.1)	6 (33.3)	
8-10	43 (67.2)	11 (61.1)	
5 min Apgar score, n (%)			
0-3	0 (0)	0(0)	0.630
4-7	2 (3.1)	1 (5.6)	
8-10	62 (96.9)	17 (94.4)	

“*”indicates that data were not normally distributed and are expressed as median (interquartile range), M(Q). Non-parametric tests were used for between-group comparisons; the normally distributed data are expressed as mean (standard deviation), $\bar{X}\pm \text{SD}.$

One-way analysis of variance was used for between-group comparisons. Chi-squared tests were utilized to analyze count data.

“#”indicates the difference was statistically significant.

POF, persistent organ failure; TOF, transient organ failure.

### Independent Risk Factors for POF Development in APIP Patients

To explore the laboratory parameters that can predict POF, the laboratory test indexes of the two group patients within 24h of admission were compared. The concentrations of triglycerides (TG), cholesterol, fast blood glucose, serum calcium, C reactive protein (CRP), aspartate aminotransferase (AST), serum creatinine (SCr), blood urea nitrogen (BUN), lactate dehydrogenase (LDH), and procalcitonin (PCT) in the POF group were significantly different from those in TOF group (P<0.05, **Table 4**).

**Table 4:** Comparison of laboratory parameters between APIP patients in the POF and TOF groups within 24 h of admission.

	TOF(n=84) (n=81)	POF(n=47) (n=23)	*P* value
WBC, 109/L	15.8 ± 4.9	14.5 ± 5.7	0.173
Hematocrit	34.2± 5.6	35.5 ± 9.5	0.418
*D-dimer, μg/L	2014 (1106-3495)	2653 (1582-4925)	0.089
*Platelets, g/L	227 (184-257)	228 (153-328)	0.905
*TG, mmol/L	8.7 (2.8-28.9)	25.0 (7.2-45)	0.003^#^
*Cholesterol, mmol/L	7.9 (4.6-15.4)	15.6 (7.1-20.7)	0.003^#^
*HDL, mmol/L	1.3 (0.9-1.6)	1.4 (0.8-1.9)	0.195
*LDL, mmol/L	2.1 (1.4-3.4)	2.2 (1.2-4.0)	0.751
APO-A1, g/L	1.3 (0.9-1.7)	1.1 (0.7-1.7)	0.169
APO-B, g/L	1.0 (0.6-1.4)	0.9 (0.5-1.6)	0.649
Na+, mmol/L	132.7 ± 8.5	130.5 ± 7.5	0.147
*K+, mmol/L	3.9 (3.5-4.1)	3.9 (3.5-4.3)	0.818
*Ca2+, mmol/L	2.05 (1.86-2.27)	1.89 (1.53-2.15)	0.015^#^
*FBG, mmol/L	7.5 (5.8-10.8)	9.2 (6.2-15.9)	0.016^#^
*ALT, U/L	11 (7-20)	19 (7-56)	0.114
*AST, U/L	21 (15-34)	38 (17-62)	0.034^#^
*Albumin, g/L	33.9 ± 35.0	28.3 ± 7.5	0.107
*Total bilirubin, μmol/L	9.4 (5.6-16.9)	6.1 (4.2-10.5)	0.052
*BUN, mmol/L	3.1 (2.3-3.8)	4.5 (2.8-8.3)	0.002^#^
*SCr, μmol/L	42.4 (37.9-51.2)	58 (44-105)	<0.05^#^
*CRP, mg/L	113 (33-189)	171 (70-328)	0.006^#^
*Blood amylase, U/L	313 (125-578)	316 (121-812)	0.882
*Blood lipase, U/L	536 (204-1020)	514 (148-1087)	0.728
*Urinary amylase, U/L	1116 (633-2521)	1427 (217-2678)	0.695
*PCT, ng/ml	0.20 (0.09-0.62)	0.74 (0.20-2.62)	<0.05^#^
*LDH, U/L	260 (218-368)	419 (292-575)	0.001^#^

“*”indicates that data were not normally distributed and are expressed as median (interquartile range), M(Q). Non-parametric tests were used for between-group comparisons; the normally distributed data are expressed as mean (standard deviation), $\bar{X}\pm \text{SD}.$

One-way analysis of variance was used for between-group comparisons.”#” indicates P**<**0.05, indicating that the difference was statistically significant.

ALT, alanine aminotransferase; AST, aspartate aminotransferase; APO, apolipoprotein; BUN, blood urea nitrogen; CRP, C reactive protein; FBG, fast blood glucose; HDL, high density lipoprotein; LDH, lactate dehydrogenase; LDL, low density lipoprotein; PCT, procalcitonin; POF, persistent organ failure; SAPIP, severe acute pancreatitis in pregnancy; SCr, serum creatinine; TG, triglycerides; TOF, transient organ failure; WBC, while blood cell count.

Clinical indicators with statistical differences (P<0.05) were included in the univariate analysis based on the results in **Table 1** and **Table 4**. The concentrations of TG, cholesterol, fast blood glucose, SCr, LDH, PCT, and trimester of pregnancy were significantly associated with POF in univariate analysis (**Table 5**). We performed multivariate logistic regression analysis with these associated factors using a stepwise method. TG, SCr, LDH, and PCT were identified as independent risk factors (**Table 5**). Furthermore, the cutoff values of the four biomarkers were calculated for predicting POF under the ROC curve (**Table 6**).

**Table 5:** Univariate and multivariate logistic regression analysis of risk factors associated with POF.

	Univariate analysis		Multivariate analysis	
factor	OR (95% CI)	*P* value	OR (95% CI)	*P* value
Age, years	1.035 (0.943-1.136)	0.471		
TG, mmol/L	1.018 (1.003-1.033)	0.016^#^	1.024 (1.003-1.024)
Cholesterol, mmol/L	1.070 (1.017-1.127)	0.010^#^		
BUN, mmol/L	1.163 (0.915-1.478)	0.217		
CRP, mg/L	1.001 (0.998-1.004)	0.506		
AST, U/L	0.997 (0.992-1.003)	0.359		
FBG, mmol/L	1.115 (1.012-1.230)	0.028^#^		
Ca2+, mmol/L	0.618 (0.161-2.367)	0.482		
PCT, ng/ml	2.600 (1.597-4.234)	<0.05^#^	2.124 (1.170-3.851)	0.013^#^
LDH, U/L	1.004 (1.002-1.007)	0.001^#^	1.006 (1.002-1.009)	0.003^#^
SCr, μmol/L	1.024 (1.009-1.040)	0.002^#^	1.026 (1.006-1.046)	0.010^#^
Trimester of pregnancy,n				
First (before14 weeks)	–	0.053		
Second (14-27+6weeks)	2.206 (0.134-36.321)	0.580		
Third (after 28 weeks)	3.309 (1.239-8.835)	0.017^#^		

“#”indicates the predictors were statistically significant.

AST, aspartate aminotransferase; BUN, blood urea nitrogen; CI, confidence interval; CRP, C reactive protein; FBG, fast blood glucose; LDH, lactate dehydrogenase; PCT, procalcitonin; POF, persistent organ failure; SCr, serum creatinine; TG, triglycerides.

**Table 6:** The cut off values of TG, Scr, PCT, and LDH for POF under the receiver operating characteristic curve.

	AUC (95% CI)	*P* value	Sensitivity	Specificity	cut-off value
TG, mmol/L	0.680 (0.575-0.785)	0.002	0.561	0.791	23.91
PCT, ng/ml	0.723 (0.624-0.822)	<0.05	0.366	0.97	1.84
LDH, U/L	0.732 (0.627-0.838)	<0.05	0.634	0.851	383.5
SCr, μmol/L	0.719 (0.614-0.824)	<0.05	0.732	0.642	47.8

CI, confidence interval; LDH, lactate dehydrogenase; PCT, procalcitonin; POF, persistent organ failure; SCr, serum creatinine; TG, triglycerides.

### Nomogram Model for POF

**Figure 2:**
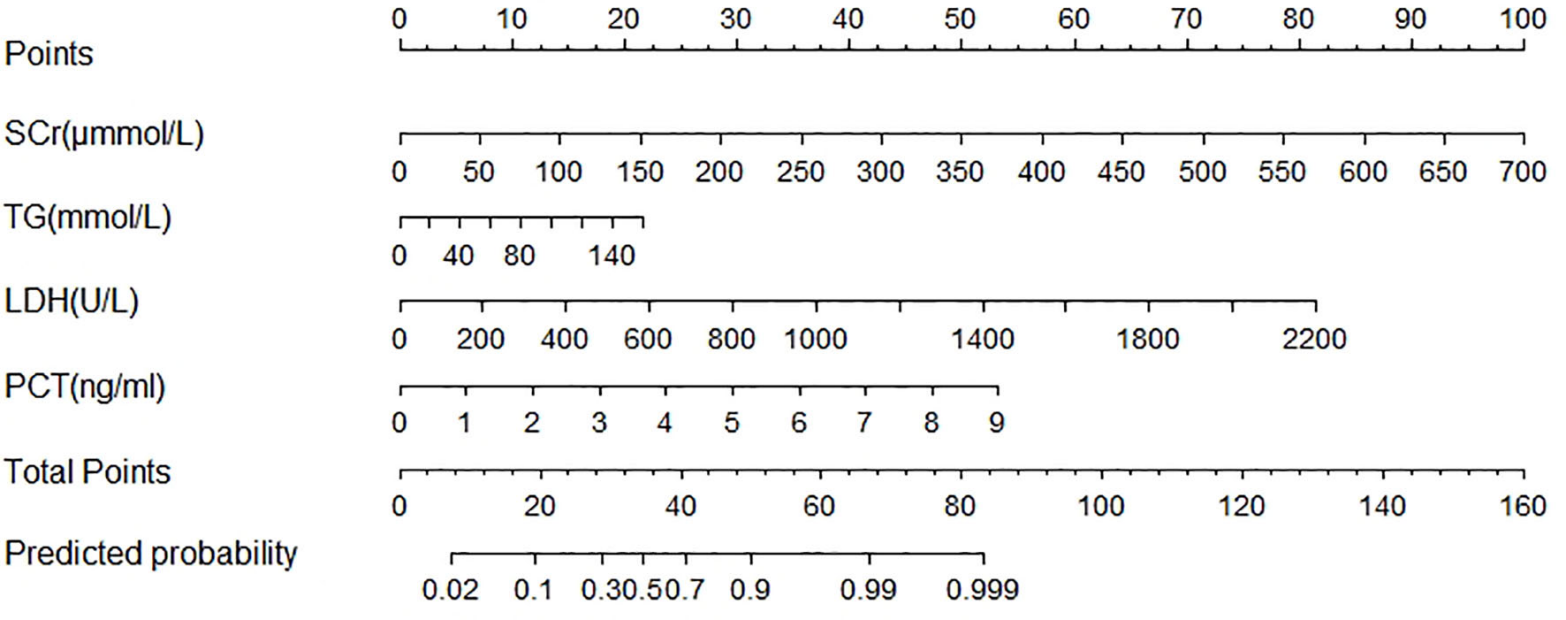
Nomogram predicted the probability of Persistent Organ Failure in patients with acute pancreatitis during pregnancy. To estimate the probability of persistent organ failure, mark patient values at each axis, draw a straight line perpendicular to the point axis, and sum the points for all variables. Next, mark the sum on the total point axis and draw a straight line perpendicular to the probability axis. LDH, lactate dehydrogenase; PCT, procalcitonin; SCr, serum creatinine; TG, triglycerides.

The POF predicting nomogram was constructed based on 4 independent predictors (**Figure 2**). We could get each point for every variable by drawing a line from each predictor straight upwards the point axis. Total point was obtained by summing up esch point. The incidence of POF could be estimated by drawing a line straight downwards the total point axis. The nomogram could be applied to estimate the incidence of POF for each patient in clinical practice.

**Figure 3:**
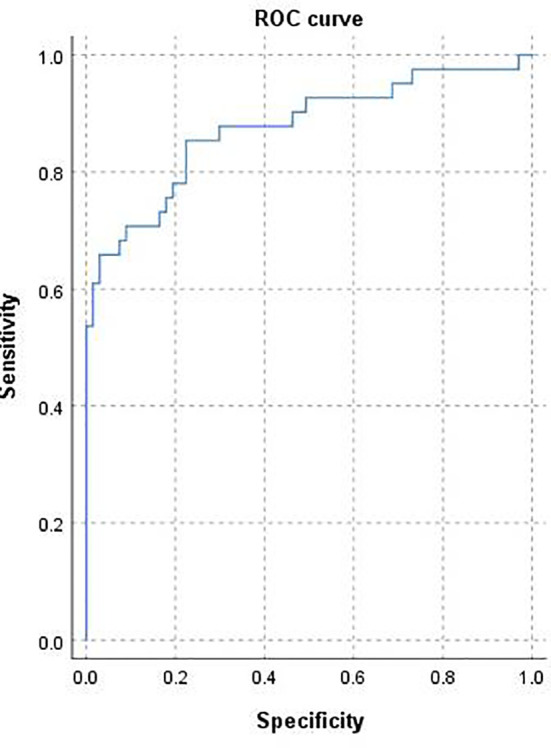
Receiver operating characteristic curve for the prediction model. Area under the curve was 0.875 (95% confidence interval 0.80–0.95).

**Figure 4:**
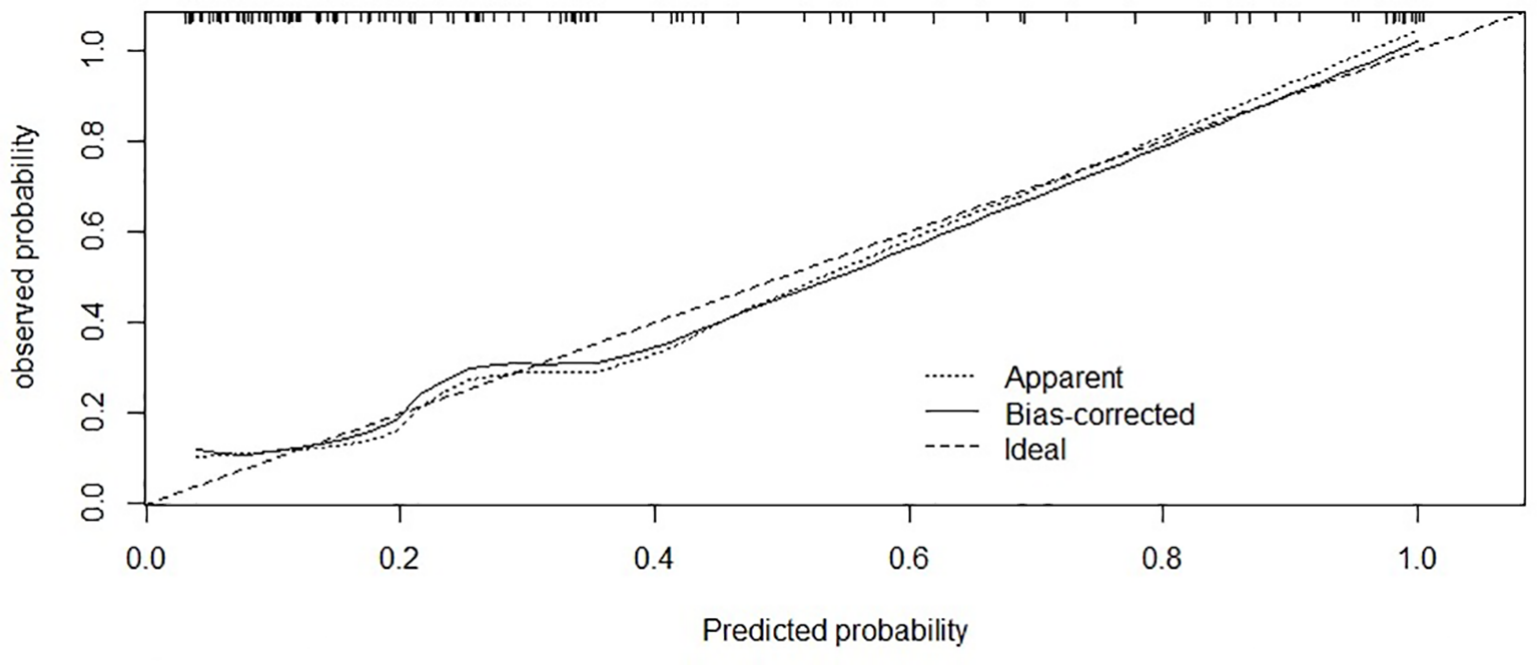
Calibration of the nomogram for risk of POF. The x-axis shows the predicted probability of POF, and the y-axis shows the observed probability of POF. C-index:0.85; (95% confidence interval 0.77-0.93).

The area under the curve (AUC) was 0.875 [95% confidence interval (CI) 0.80–0.95; **Figure 3**] and the concordance index [C-index) of the nomogram was 0.85 (95% CI, 0.77-0.93; **Figure 4**), indicating that the nomogram was accurate in estimating the risk of POF. The calibration of the model was also assessed with calibration curves. **Figure 4** shows that predictions made by the model were close to the observed outcomes, which further demonstrates the reliability of the nomogram in POF risk estimation.

## Discussion

This study demonstrated that TG, Scr, PCT, and LDH are the independent predictors and developed a user-friendly nomogram with clinical usefulness to predict the individual probability of POF in APIP patients. To our knowledge, this study is the first attempt to establish a prognostic nomogram model for POF based on the clinical data of APIP patients within 24h of admission. The four indicators can serve as early biomarkers to differentiate POF from TOF in APIP patients.

HTG is the third most common cause of acute pancreatitis, accounting for 4-10% of cases in the general population (19). Many previous studies reported that the risk of severe acute pancreatitis and mortality were higher in the hypertriglyceridemia group compared with other causes of the disease (20–22), while no correlation between the admission triglyceride concentration and the severity or the morbidity of those HTGP patients were found (23). Our previous study analyzed a group of APIP patients (24). We found that when the admission TG was less than 7mmol/L, the possibility of SAP decreased with the TG level, while when it was more than 7mmol/L, the possibility of SAP increased with the TG level. The cut-off value of the admission triglyceride concentration for predicting the occurrence of SAP is 10.7mmol/L, the sensitivity is 0.72, and the specificity is 0.65. In the present study, we found that TG was an independent risk factor in prediction of POF in APIP.

Serum creatinine (Scr) is a marker of renal function. A rise in Scr reflects the disease states of initial hypovolemia and renal dysfunction in SAP and it represents an important factor for the assessment of severity (25).

Procalcitonin is the precursor of the hormone calcitonin found in the thyroid C cells and the pulmonary endocrine cells (26). However, all tissues have the potential to produce procalcitonin (27). During systemic infections, blood PCT levels rapidly increase within 3–6 h, persisting during the inflammatory process, and decrease as the infection starts to subside (28). Reza has reported that serum PCT was a valuable predictor of SAP in the general population (29). Hence, PCT may facilitate the monitoring of progression in AP in general people (29–31).

LDH is an indicator of cell death. Since pancreatic necrosis is associated with cell death, LDH may distinguish between oedematous pancreatitis and pancreatic necrosis. As systems for evaluating the severity of AP, both Ranson and JSS systems enrolled LDH in their scoring criteria (32). In studies of non-pregnant populations, LDH has been validated as an independent risk factor for predicting AP severity.

The predictive nomograms are derived based on existing clinical data and provide a visible and easy-to-use ruler for the predicted probabilities: the results can be generated very quickly and reliably by drawing only one or more lines without replacing the numbers into the equation (33). Our predictive model was generated from parameters within 24 hours of admission, which used a series of convenient and inexpensive laboratory values that could be routinely utilized in the clinical setting, and was applicable in a user-friendly manner.

Some previous studies found that decreased HDL had predictive value for SAP in the general population (12, 25). However, in this study, we didn’t find any difference in HDL level between the POF and the TOF groups, which could be due to the different etiological characteristics of AP in the general population and APIP. The etiology of AP in the general population is mainly biliary type (34, 35), while APIP is mainly HTG type (1). The changes of lipid metabolism in the latter may be different from that in biliary type AP. In addition, increased estrogen during pregnancy can cause an increase of HDL during gestation (4), which could also explain why no difference in HDL level between the two groups was found in this study.

We also observed that compared with the TOF group, the POF group had older average age, the etiology of HTG was also higher in the POF group. These findings suggest that in future clinical work, more attention should be paid to the onset of gestation in older and hypertriglyceridemic patients with APIP.

In our study, all 7 maternal death was in the POF group (14.89%). And more fetal loss were observed in the POF group than in the TOF group (61.7% VS 22.6%, P<0.05, **Table 2**). This is similar to previous reports of Luo et al. (15). In their study of 121 APIP patients, the mortality rate of pregnant women in the SAPIP group was up to 22.2%. The fetal loss rate was 44.4% and 9.1% in the SAP and MSAP groups respectively. These results suggest that POF in APIP is a serious condition with an increased risk of adverse maternal and infant outcomes.

Through the establishment of a nomogram prediction model, a prediction tool with simple operation is provided. The probability of POF in APIP can be obtained easily by using a series of convenient and cheap laboratory values that could be routinely utilized in a clinical setting within 24 hours of admission.

In the present study, we retrospectively analyzed the APIP patients with OF in a tertiary medical center of Northeast China in the last 9 years, 131 singleton APIP patients with OF were enrolled in our research. It was the largest OF-related APIP study to date.

Our study also had some limitations. Firstly, as a cross-sectional study, due to the low incidence of SAPIP and the limited number of related samples, the prediction model established in this study needs to be further validated. Secondly, the study samples were collected in China, therefore the results lacked representation of the non-Chinese population. Therefore, whether the results of this study can be applied to other ethnic groups warrants further investigations.

## Conclusions

In conclusion, we established a useful nomogram model with easily obtainable biochemical parameters. This laboratory-based model allows practitioners at the bedside for early risk-stratification for POF with APIP patients. Early identification of patients with possible POF in APIP is of great significance in improving prognosis.

## Data Availability Statement

The original contributions presented in the study are included in the article/supplementary material. Further inquiries can be directed to the corresponding author.

## Ethics Statement

The studies involving human participants were reviewed and approved by the ethics committee of the Shengjing Hospital of China Medical University (Approval No 2019PS683K). The ethics committee waived the requirement of written informed consent for participation.

## Author Contributions

All authors conceived and designed this study. CS, JW, and ZX interviewed participants and collected clinical data. CS and JW analysed the data with relevant statistics and provided discussion points in the study. JW reviewed and provided crucial interpretation of study results. All authors contributed to the writing of this paper but CS and JW edited the final version. JW is the corresponding author. All authors contributed to the article and approved the submitted version.

## Funding

This study were supported by Natural Science Foundation of the Liaoning Province (20180530076).

## Conflict of Interest

The authors declare that the research was conducted in the absence of any commercial or financial relationships that could be construed as a potential conflict of interest.

## Publisher’s Note

All claims expressed in this article are solely those of the authors and do not necessarily represent those of their affiliated organizations, or those of the publisher, the editors and the reviewers. Any product that may be evaluated in this article, or claim that may be made by its manufacturer, is not guaranteed or endorsed by the publisher.
